# mpactR: an R adaptation of the metabolomics peak analysis computational tool (MPACT) for use in reproducible data analysis pipelines

**DOI:** 10.1128/mra.00997-24

**Published:** 2025-01-15

**Authors:** Allison R. Mason, Gregory Johnson, Jr, Joseph Krampen, Jennifer N. T. Nguyen, Marcy J. Balunas, Patrick D. Schloss

**Affiliations:** 1 Department of Microbiology & Immunology, University of Michigan, Ann Arbor, Michigan, USA; 2 Department of Medicinal Chemistry, University of Michigan, Ann Arbor, Michigan, USA; Loyola University Chicago, Chicago, Illinois, USA

**Keywords:** metabolomics, software, R package, data curation

## Abstract

mpactR automates pre-processing of liquid chromatography-tandem mass spectrometry (LC-MS/MS) data from microbiological samples to correct mispicked peaks, resolve inter-sample variation in abundance across technical replicates, account for in-source ion fragmentation, and remove background noise to yield high-quality mass spectrometry features. The package is available through CRAN and GitHub.

## ANNOUNCEMENT

There has been increased interest in performing metabolomic analyses of microbial communities with the goals of understanding the relationship between the structure and function of these communities and linking changes in the metabolome with changes in health. Combined with DNA and RNA-based sequencing of microbiomes, the broader availability of liquid chromatography-tandem mass spectrometry (LC-MS/MS) has made untargeted metabolomics a powerful addition to multi-omics analyses (e.g., 1–3). Traditionally, LC-MS/MS has been used to identify novel bioactive natural products. Incorporating metabolomics into microbiome studies presents several novel challenges. These include accessing software that can process large numbers of complex biological samples. Although there are numerous options for analyzing LC-MS/MS data, the software tends to be expensive, proprietary, has poor interoperability and is accessible only through a graphical user interface (GUI) as a website or stand-alone application. Additionally, analysis of large metabolomics data sets is complicated by inherent data variability. These factors limit the ability to foster transparency and reproducibility.

Initial efforts to overcome these challenges have included packages including the tidymass ensemble of R packages (4) and the metabolomics peak analysis computational tool (MPACT) (5). The tidymass ensemble is an open source R package seeking to centralize the full data workflow within the R ecosystem. However, existing peak-picking algorithms are error-prone, reducing our ability to identify high-quality ions. MPACT is an open source Python package allowing researchers to reconcile mispicked peaks, resolve inter-sample variation in abundance across technical replicates, account for in-source ion fragmentation, and remove background noise using data collected from solvent and/or media blanks. MPACT can only be used as a GUI and has limited options for visualizing and analyzing the processed data; therefore, we developed mpactR as an open source R package that provides users the useful functionality of MPACT while integrating the processed data in a manner that satisfies their research needs using the R ecosystem.

mpactR’s website provides detailed installation instructions to install the package (https://www.mums2.org/mpactr/) from the Comprehensive R Archive Network (CRAN) or GitHub. The website provides tutorials on how to use the mpactR package as well as tutorials for using R with the output of mpactR to generate the types of figures that were originally produced in MPACT. For consistency, these tutorials use an example data set characterizing the metabolome of the tunicate-associated bacterium *Streptomyces* sp. PTY087I2 used in the original MPACT publication (5). mpactR is designed using the R6 object-oriented programming system with reference semantics, allowing for minimal memory footprint while executing at least as fast as MPACT.

mpactR was designed to be straightforward to use and easy to integrate in automated reproducible analysis pipelines. mpactR requires two comma-separated values files, including a peak table and a metadata file containing relevant sample information that can be read in using the import_data function. Peak tables from multiple upstream analysis tools are accepted, including but not limited to Progenesis and Metabocape. Instructions on how to generate these files are provided at the mpactR website. Filtering is performed using any of four functions, and the package provides four summary functions to allow users to evaluate the effects of filtering. The accessor returns the processed data in the data.table package format facilitating rapid and memory-efficient analyses with other data.table functions, used with functions from R packages in the tidyverse, including ggplot2, or written to a file for uploading to the GNPS website (6).

## Data Availability

Stable releases of mpactR are available through CRAN and developmental versions are available through the project’s GitHub website (https://github.com/mums2/mpactr). The package is available under the MIT Open Source License.
